# Participant Experiences in a Human Biomonitoring Study: Follow-Up Interviews with Participants of the Flemish Environment and Health Study

**DOI:** 10.3390/toxics9040069

**Published:** 2021-03-28

**Authors:** Bert Morrens, Hans Jonker, Elly Den Hond, Dries Coertjens, Ann Colles, Greet Schoeters, Nicolas Van Larebeke, Tim Nawrot, Adrian Covaci, Vera Nelen, Frédéric Vandermoere, Ilse Loots

**Affiliations:** 1 Faculty of Social Sciences, University of Antwerp, 2000 Antwerp, Belgium; hans.jonker@uantwerpen.be (H.J.); dries.coertjens@uantwerpen.be (D.C.); frederic.vandermoere@uantwerpen.be (F.V.); ilse.loots@uantwerpen.be (I.L.); 2 Department of Health, Provincial Institute of Hygiene, 2000 Antwerp, Belgium; elly.denhond@provincieantwerpen.be (E.D.H.); vera.nelen@provincieantwerpen.be (V.N.); 3 Health Unit, Flemish Institute for Technological Research (VITO), 2400 Mol, Belgium; ann.colles@vito.be (A.C.); greet.schoeters@vito.be (G.S.); 4 Department of Radiotherapy and Nuclear Medicine, University Ghent, 9000 Ghent, Belgium; nicolas.vanlarebeke@ugent.be; 5 Department of Analytical, Environmental and Geo-Chemistry, Vrije Universiteit Brussel, 1000 Brussel, Belgium; 6 Center for Environmental Sciences, Hasselt University, 3500 Hasselt, Belgium; tim.nawrot@uhasselt.be; 7 Toxicological Centre, University of Antwerp, 2610 Wilrijk-Antwerp, Belgium; adrian.covaci@uantwerpen.be

**Keywords:** human biomonitoring, risk communication, research participation, environmental health, report-back, participant experiences

## Abstract

Communicating individual human biomonitoring results to study participants has been the subject of debate for some time. This debate is dominated by ethical considerations from a researchers’ perspective on whether or not to communicate, thereby overlooking more practice-based questions from a participants’ perspective on what and how to communicate. We conducted a small scale follow-up study based on eleven face-to-face interviews with mothers participating in the third cycle of the Flemish Environment and Health Study (FLEHS III 2012–2015) to investigate how they experienced and interpreted individual biomonitoring results. Key findings indicate that respondents were generally satisfied with participating in the biomonitoring study, but the report-back process especially lacked contextualized information and interactive communication options to better comprehend and cope with personal results. These findings also argue in favor of a more tailored approach in which report-back methods, formats and content are diversified according to the type of results and the preferences of participants. A reflexive research practice with active engagement in follow-up research is crucial to improve participants’ understanding and use of personal biomonitoring results.

## 1. Introduction

Human biomonitoring (HBM) measures the concentrations of environmental chemicals, their metabolites and their biological responses in body fluids and tissues, such as blood, urine, breast milk, hair or nails [1]. HBM is a subdiscipline of molecular epidemiology which can contribute importantly to a better knowledge of chemical exposures and of environmental causes and risk factors for disease. Over the past decade, the number of national or regional HBM-programmes has increased and is often surveillance-based, to monitor nationwide or regional reference ranges of internal concentrations to an increasing number of chemicals, ranging from historic pollution, such as persistent pesticides and toxic metals, to new emerging substances used in plastics, cosmetics, furniture, and other consumer products [2,3]. HBM is also used in a variety of other settings, such as community-based biomonitoring studies, to respond to local concerns about environmental health risks, or advocacy work from civil society organizations [4,5].

HBM studies most commonly report statistical results and interpretation of aggregated data from (large) study samples. In addition, HBM can also generate personal result reports containing the measured biomarker concentrations of an individual, often accompanied by some comparison to reference or population values. However, there is no universal consensus nor common practice to communicate individual results to study participants [6,7]. In fact, there is considerable debate on this topic, making report-back of individual results one of the main challenges of HBM today [4,6,8]. This debate is often reduced to a principle-based ethical dilemma on whether or not to communicate personal results to study participants [9]. On the one hand, the clinical ethics perspective emphasizes report-back only if concentrations exceed clinical action levels or if the relationship between biomarker levels and health risk is clearly understood. Traditionally, most surveillance-based biomonitoring studies adopt this clinical ethics approach and communicate results only at an aggregated level [5,8]. On the other hand, the community-based participatory research (CBPR) approach emphasizes extensive reporting back of personal and aggregated results. In this framework, results should be disseminated to participants not primarily because they have a clinical significance, but rather for its preventative or precautionary significance: to motivate behavior change, to sensitize about environmental health risks and to increase trust between participants and scientists [10,11]. The CBPR approach is mostly applied in local biomonitoring studies in the US, often within disadvantaged communities in an environmental justice context [12], yet some European surveillance-based HBM studies integrate principles of the CBPR approach in their communication strategy [8,13,14,15].

However, this ethical discussion omits several more practice-based questions and challenges about how to communicate individual results in a responsible and meaningful way. In general, participants want to know their results, but only reporting numerical concentrations of chemicals is deemed not very useful to lay people. Follow-up studies on participant’s experiences advocate for a more extensive contextual framework to guide participants with the interpretation of their personal data [16,17]. Brody, et al. [18] for example have identified typical participants’ questions about HBM results that go beyond a mere list of chemicals detected, such as “is it safe?”, “what should I focus on?”, and “what can I do?”. These questions emphasize the importance of understanding how participants process, interpret and respond to the presence of bodily contaminants, a concept which has been termed “the exposure experience” [19,20]. Participants are not considered as just passive receivers that “get the message”, but as active actors that understand and interpret messages according to their past experiences, attitudes and perceptions [21]. Based on this contextual framework, Brody et al. [22] and Dunagan et al. [23] have documented detailed and practical guidelines for communicating personal results to participants. These guidelines, however, are mainly based on experiences from specific community-based studies in the US that use a bottom-up recruitment strategy and study design. Less knowledge and practice is available on national surveillance-based human biomonitoring programs that include randomly selected participants in a more top-down approach, on behalf of a public authority. In this context, the objective to communicate is usually focused on transparency and health promotion, rather than advocacy. In addition, surveillance biomonitoring may experience fewer opportunities to mobilize participants around a shared local concern, and a greater distance between researchers and participants, which challenges meaningful report-back. In this article, we want to investigate how study participants of a surveillance-based human biomonitoring study in Flanders (Belgium) experienced and interpreted their individual biomonitoring results. We conducted a small scale follow-up study based on face-to-face interviews with mothers that participated in the Flemish Environment and Health Study (FLEHS). Results of FLEHS-studies have already been extensively reported [24,25], as well as their relevance to policy [26], but no research has yet been performed on how results are interpreted by FLEHS participants themselves. We used an explorative set-up to evaluate report-back protocols and generate hypotheses about the participants experiences in the FLEHS-study. Our study aims to provide more insight into how participants understand and interpret their biomonitoring results and to make recommendations to enhance report-back practices for large biomonitoring/surveillance studies.

## 2. Materials and Methods

### 2.1. FLEHS: Flemish Biomonitoring Study of Mothers and New-Borns

In Flanders, the northern part of Belgium, the human biomonitoring program FLEHS has been established since 2001 to measure and monitor internal concentrations of environmental pollutants (biomarkers of exposure) and associated biological effects (biomarkers of effect) in different age groups of the Flemish population [24]. The study is commissioned by the regional Flemish government and carried out by a multi-disciplinary research consortium. The primary objectives are to generate reference values for a diverse set of biomarkers, to follow-up time trends and study exposure determinants and exposure-effect associations. A more detailed description of the FLEHS study design is available elsewhere [27].

For our follow-up study, we focused on a cross-sectional study with 281 mothers and new-borns carried out between 2013 and 2015, within the third FLEHS-cycle. The aim was to measure internal exposure to hazardous chemicals in a geographically representative sample of the Flemish population. For this HBM-study, women were recruited in five randomly selected maternity hospitals in each of the Flemish provinces. Midwives informed pregnant women about the study and invited them to participate when they registered in the maternity for delivery. All women who resided for at least five years in Flanders were eligible for study participation. Study nurses explained the research protocol in detail on the basis of a brochure and invited them to sign the informed consent form. All participants donated cord blood and consented to take a biopsy from the placenta. Optionally, they agreed to donate a hair sample (collected in the maternity by the study nurse) or a finger nail sample (self-collected for four weeks from the day of delivery). In the days after delivery, the mothers completed an extensive questionnaire (self-administered) providing information on lifestyle, health status, food consumption, use of tobacco and alcohol, residence history, education and occupation. In the biological samples, biomarkers of exposure such as toxic metals, persistent organochlorinated pollutants, brominated flame retardants and perfluorinated compounds, and biomarkers of effect, such as hormone levels and markers of DNA damage, were measured by specialized labs.

### 2.2. Communication Strategy and Report-Back Protocol

Within the FLEHS program, a detailed and transparent communication strategy has been developed [14], including a report-back protocol of individual biomonitoring results (based on participants’ right to know) and a sequenced communication of research results at a group level in which participants are informed prior to the general public (“participants first” principle). Participants received their individual results by post at their home address at the end of 2015, if they had chosen this option in the informed consent (91.8% of participants did). Undelivered mails were returned to the research institute in case of a move or incorrect address, otherwise we assumed that all participants received their letter well. Together with an introductory letter (see Supplementary Material), a table format (see Table S1) was used to present results for seventeen biomarkers of exposure. Table 1 below illustrates this data format for three biomarkers.

Individual results for each biomarker were compared with two reference values, i.e., the median and 90th percentile (P90) of all study participants. No health-based guidance values were included since these were not available for cord blood levels at that time. Contact information of the study physician was added in the letter for participants who wanted a personal consultation at two predetermined moments. There was an early notification protocol, in case of high level results that required further follow-up (values with clinical significance or extreme outliers). The study physician would then personally call participants prior to sending the results. This protocol was described in the introductory letter.

Participants also received a document with background information on each pollutant (see Table S2). The document contained information about pollution sources, exposure routes and possible health effects. An illustration is found in Table 2. A referral to the study website was included for exposure reduction advice. Along with the personal results and background information, participants received a one-page summary of the general research conclusion, based on the aggregated results.

All documents were developed in the field work committee by a multidisciplinary research consortium, taking into account previous experience of the research and input from earlier participant reviews since this was the third cycle of the Flemish HBM program.

### 2.3. Follow-Up Interviews

In March 2018, participants were re-contacted and invited to participate in a personal follow-up interview to discuss their experiences after receiving their biomonitoring results. For practical reasons, invitations were restricted to 85 participants living in the province of Antwerp. They received a written invitation at their home address and could register by email or phone. Follow-up interviews were held at the University of Antwerp or at their home address in April and May 2018. Interviews were recorded with permission. The interview protocol used open-ended questions and consisted of three parts: study participation (how did respondents experience the study?), study results (how did they understand their results?) and study impact (how did they respond to their results?). The interview guideline is included as a supplemented file. All interviews were conducted in Dutch by the corresponding author. Interview audios were transcribed, and answers were categorized into relevant themes and issues.

## 3. Results

Out of 85 invited participants, 11 were willing to cooperate (13% participation rate). Taking the participant’s preferences into account, seven interviews took place at the respondent’s home, and four at the University. The median interview length was 56 min (range: 28–60 min). Demographic and socioeconomic characteristics of the eleven respondents of the follow-up interview are presented in Table 3. Eight respondents were between 25 and 35 years of age. Eight respondents had a high educational attainment (tertiary education), three had a medium educational attainment (secondary education). Nine respondents were gainfully employed, one was unemployed and one was a housewife. Four respondents have a migrant background, defined as having at least one parent that was not a native-born Belgian. Compared to the study sample of HBM participants (Table 3), the respondents of the follow-up interview had a higher rate of tertiary education, a high income and home ownership, yet they also had a higher unemployment rate and migrant background status. The age distribution and parity of respondents and participants were comparable. This small sample is not representative of the total participant group nor the Flemish population, nevertheless it includes some meaningful differences in the participant profiles.

### 3.1. Study Participation: How Did Respondents Experience the Study?

Despite the long time span of three years between study recruitment and follow-up interviews, nine respondents remembered that the reason for participating in the biomonitoring study was because they valued the research topic of environmental health and environmental pollution or because they wanted to make a contribution to science (Table 4). For the five respondents, an additional trigger for participation was the fact that they would receive individual results. This was articulated in terms of curiosity to know their body burden and not because of a health concern. Moreover, for two women, the motivation to participate was based on the conviction and expectation that their individual results would be good, because it would reflect their healthy lifestyle or their personal living environment, which they perceived as being safe.

None of the respondents perceived the legal language in the informed consent as a barrier to participate. Most women immediately agreed to participate and did not need time for consideration.

Half of the respondents did not recall reading the information brochure of the study. The other half mentioned that, although the content of the brochure was professional and accessible, the framing was too medical and scientific, which created a mental distance between them and the researchers. For example, the technical description of the different biological samples that would be collected remained abstract. Because the brochure did not mention why these different samples were needed, mothers felt somewhat detached from the broader study objectives and were a bit cautious about donating their samples, especially the hair sample.

“The placenta, that’s normal. That’s what the research is all about. But hair? This is perhaps strange to say, but I can remember that at that moment I felt like: “that is mine”. They ask for a lock of my hair... that was weird”.

(R7)

Around half of the respondents indicated that the self-administered questionnaire was long and contained many questions. Yet the interviewed women realized that human biomonitoring research inevitably requires a lot of information. Moreover, respondents did not find the questions too difficult or too sensitive to complete. Interestingly, one mother projected certain questions on their personal situation, to make assumptions on risk behavior and exposure determinants, for instance about the use of indoor spray products.

### 3.2. Receiving Results: How Did Respondents Understand Their Results?

When asked about the initial feeling when receiving individual results, four respondents felt confused about their results and indicated the need for additional information about health implications (Table 5). Firstly, participants not only wanted reference values to compare their results with other study participants, they also wanted health-based guidance values to interpret their exposure levels in safety terms. Secondly, several mothers expected more information and an interpretation of the extent to which their results reflected a transfer of contamination to their baby and potential health effects. In addition to information to evaluate and interpret health risks, the mothers suggested using a visual summary that could guide them through the multitude of results. For instance, by adding an overview with some key takeaway messages, or by transforming the enlisted result tables in a graphical format like a bar chart.

Women with a high biomarker result (*n* = 5) expressed a feeling of concern that often overwhelmed them at first. One mother remembered:

“I just looked at the deviated values, and lead was very high for me. That did make me feel anxious. Because that was tremendously different from the rest and then I got totally fixated on that”.

(R9)

Despite these initial feelings of concern or confusion, none of the respondents mentioned anxiety or panic, in fact only one mother actually phoned the study physician for more information. However, four of the mothers indicated that they did not remember this option or that they found the wording in the letter not inviting or accessible enough. Three other respondents described a certain reluctance to call because they perceived the study to be a large-scale scientific study and not an individual follow-up. They felt that asking personal questions would interrupt the study team and would go beyond the scope of the study. The statement of the early notification by the study team in case of medical significance further contributed to the mothers reluctance to call:

“One of those values was really high for me, especially compared to all the rest. And... that was disturbing, personally [...]. Of course, the letter mentioned that if you had any questions you could contact them... and uhm... It’s weird, I’ve always had that in mind: once, I’m going to call for that, I want to know: what could it be? […] But it never really happened and from the research itself, I never heard anything... And at a certain point I made the assumption: no, if it was really bad they would notify me”.

(R7)

When asked about the usefulness of a face-to-face consultation with the study physician instead of a phone call, three women felt this would have been a better approach to deal with their questions. The supplemented table with background information about the measured pollutants (see Table S2) was designed to help participants understand and interpret their results. Nevertheless, the majority of respondents considered the information to be too generic and too abstract to be helpful. The lack of practical advice and solutions to reduce exposure especially made the table of limited use for participants. According to four respondents, the focus was on a scientific explanation of the different pollutant sources and exposure routes, thereby ignoring tailored and practical solutions for exposure reduction.

Furthermore, the list of possible health effects in the background table triggered a feeling of unease among two respondents, because it did not indicate a threshold exposure level above which these effects can be expected. Six mothers did not remember receiving or reading the summary of the aggregated research results. The other mothers referred to the fact that the summary contained too much text, was too difficult or too generally written.

### 3.3. Study Impact: How Did Participants Respond to Their Results?

Because most respondents indicated having insufficient contextual information to interpret their results, some searched for additional information on the internet (n = 3). Only three mothers took more extensive action (Table 6). One participant had a sample of her drinking water analyzed to determine the source of her elevated value of lead. One mother went to her general practitioner to discuss the study results. Another women removed the chickens from her yard because they were possibly causing exposure to a perfluorinated compound. Six respondents did not undertake action, which appeared to be mainly because the results provided not enough tools to take effective action. As a result, information was less likely to be remembered and the importance of acting on behalf of the results quickly faded with time:

“I found this package [of results] to be primarily informative and not action-oriented. That’s how that whole package was set up and that’s how I understood it. And somewhere that is in your head and we kept it in our minds for a while, but in the hustle and bustle of everything you leave it behind and then… you have to take too many steps yourself to do something with it, I suppose”.

(R4)

Furthermore, as the results sometimes failed to resonate with mothers’ conscious knowledge and beliefs, four mothers even mentioned they actively wanted to forget or suppress their results: “I was like, I don’t want to know, just leave it like that. I admit it. Don’t think about it too much...” (R3).

## 4. Discussion

The Flemish human biomonitoring program FLEHS places a strong emphasis on comprehensive report-back of study results to participants, both on an individual and collective level. We conducted a small scale follow-up study to evaluate the report-back process of the individual results of the biomarker measurements from a participants’ perspective. Based on eleven semi-structured interviews with mothers who participated in the FLEHS III study on new-borns, we learned that these participants were generally satisfied with participating and preferred to know their own results. However, receiving results did not appear to motivate or inspire most participants to take action, nor did it enhance their knowledge of environmental health. This is probably because the report-back process lacked both contextualized information and interactive communication options. Hence respondents found the human biomonitoring results complex to understand and not really meaningful for their personal lives. The medical and scientific framing of the study during the consent and recruitment phase created a mental distance for these participants and made them reluctant to contact the research team. To compensate for this lack of contextual information and perceived study support, some respondents searched for other information to construct their own interpretation of the results, or chose to suppress or ignore their results.

Our follow-up study indicated that this knowledge construction of participants even starts early in the research process. For example, during recruitment and sampling, study documents that were deemed to be value-free and neutral, like the information brochure and the questionnaire, became part of the sensemaking process of participants. When these documents lack clear and transparent information, participants will form their own view about the study objectives. It therefore seems crucial to better explain why the various personal and intimate body samples were related to the general study objectives. According to some respondents, this should have been done with narrative information and oral communication rather than scientific information and written communication

During the report-back process, participants especially lacked health-based guidelines, a summary of key messages, and practical exposure-reduction advice with their personal results. This corresponds well with the typical participant questions identified by Brody et al. [18] To make the results meaningful, personal results should explain what is known and not known about health implications and exposure reduction [22]. However, communicating health-based guidelines and exposure reduction actions is not without difficulties. Firstly, for many pollutants, health-based guidance values are subject to scientific discussion or are simply not available [23]. Secondly, most guidelines are derived at the group level, which complicates individual health risk assessment. Thirdly, when participants do get to compare their results with guidance values, there is a risk that participants will either normalize problematic exposure results and have a false sense of safety when their score is below the guideline or cause unnecessary anxiety in case their value is above the guideline [18].

Another complexity in reporting back results is providing participants with clear and relevant exposure reduction strategies, as similar issues with health guidelines emerge. Brody et al. [18] state that recommendations for action should reflect the level of available knowledge about health effects and exposure reduction methods. Low knowledge of both factors results in recommendations for future research and more precautionary action; high knowledge of both factors results in a recommendation for public health policy and individual action. This requires combining evidence from biomonitoring studies and exposure science.

The abovementioned complexities call for a more tailor-made approach in which report-back methods, formats and content is diversified according to the type of results and the preferences of participants [28]. For instance, Rothstein [29] suggested a “tiered disclosure approach” in the recruitment phase of a study in which participants could select from options for research disclosure, for instance to be notified by a physician or by letter. Buck et al. [30] used personalized report-back protocols at the end of their biomonitoring study, by designing two versions of standardized letters depending upon participants results being either low or high. Participants with concentrations above a reference value received a “high letter” with extra guidance and additional information.

More recently, Boronow et al. [28] have used digital methods to reduce practical barriers to report-back and to tailor disclosure preferences. Their Digital Exposure Report-Back Interface (DERBI) produces personalized result reports, based on scientific input and automated decision rules. Within a digital environment, participants can access and explore their personal results more easily and effectively by using a user-centered design and personalized software generated messages, together with a combination of text and interactive graphs. By offering participants complex information using information hierarchy, which combines understandable charts, short messages and text, the understanding of participants is maximized [23]. To consult digital reports in DERBI, participants only need a computer with internet access. No software is required. A more optimal version for smartphone use is currently under development. This is an important adjustment for certain socially vulnerable groups.

Besides contextualized information, our follow-up interviews also revealed a perceived lack of opportunities to communicate and interact with the researchers. Concerning the recruitment and consent phase of the study, the interviewed women expressed the need for more face-to-face information about the study earlier in their pregnancy. This expectation of a more timely dialogue with researchers about the informed consent was also reported in other follow-up studies with mothers donating placenta samples [31]. For the report-back phase, women indicated a face-to-face consultation with the study physician would be a more accessible way to discuss results.

Interactive communication (home visits, community meetings) is an important way to build trust and understanding between scientists and participants [16,32], but is especially challenging in cross-sectional and surveillance-based HBM programmes that use a more generalist approach and often have a more extensive timeframe between recruitment and dissemination of results. In addition, a heterogeneous participant group with randomly selected volunteers from the general population is more difficult to mobilize around a specific problem or concern. When recruiting and sampling participants, study nurses invested a lot of time in personal contact to put participants at ease, and this was highly appreciated by the respondents. However, this personal contact could not be sustained until the dissemination of results. If face-to-face interaction after the recruitment phase is not feasible, creating a secure mailing list of interested participants could be helpful to maintain contact with the research team, for instance by a digital newsletter with updates of the research progress or an interactive webinar to discuss research results. This has been mentioned as an important tool to keep participants engaged throughout the study [17].

From a researcher’s perspective, interacting with participants could also be helpful in the design phase to shape study protocols and report-back materials to local and cultural needs [6,32]. This can be done by organizing an advisory committee and by pre-testing study documents with the target group [33,34].

The approximately 3 year interval between women’s initial participation in the study and the follow-up interviews is without doubt a limitation to fully capture participant experiences in a human biomonitoring study. However, respondents were generally able to recall and articulate their initial feelings and experiences during study participation and after receiving results. Similar and even longer time lags are reported in other case studies that evaluate reporting back of personal biomonitoring results [21,30]. Another limitation of this study is the limited sample size and the specific target group of mothers of newborns, which makes it difficult to generalize the results. In addition, the sample was not representative of HBM participants. Still, the study provides interesting insights and recommendations to improve report-back practices, which can be summarized as follows:

- Engage with participants in the designing phase of the study to obtain advice from the target audience and to pretest report-back materials.
- Communicate during the consent process regarding when and how research results will be returned to participants to help set appropriate expectations.
- Provide results reports in multiple formats that participants could choose from and diversify in the presentation of results.
- Define a clear general takeaway message, including practical recommendations, to reduce exposure to chemicals.
- Create opportunities to discuss results directly with researchers and other participants.

This study also contributes to describing European experiences regarding report-back of individual research results. The current literature is mainly based on US experiences, grounded in traditions of environmental justice and public sociology. These traditions have developed differently in Europe [4]. An interesting question for future research would be whether and how this affects report-back practices.

## 5. Conclusions

The current study sheds light on some critical challenges related to the communication with research participants in the context of a surveillance-based HBM study, by showing how participants experienced the study, what they learned and gained from receiving their personal HBM-results, and the type of information they need. Understanding the process and considering the sensemaking that participants experience during and after the study is of key importance when designing HBM report-back materials and protocols. We believe a reflexive research practice with active engagement in evaluative follow-up research is crucial to improve participants’ understanding and use of personal HBM-results.

## Figures and Tables

**Table 1:** Illustration of the data format used for individual report-back of HBM results for three out of seventeen biomarkers (translated from Dutch) (2013–2015).

Toxic Metals	Your Result	Results of All Participants	
		Median	P90
Cadmium (µg/L)	X *	0.021	0.034
Lead (µg/L)	Y *	6.07	11.50
Copper (µg/L)	Z *	570.95	685.61

Note * is where the personal result is shown.

**Table 2:** Illustration of background information for individual report-back of HBM results, for three out of seventeen biomarkers (translated from Dutch) (2013–2015).

Pollutants	What Are the Main Sources in Our Environment?	How Are Humans Exposed?	What Are Possible Health Risks?
Cadmiumin in blood; exposure measurement of previous 3–4 months	cigarette smoke non-ferrous industry, scrap processing industry in the past: domestic waste incinerators (e.g., battery combustion) and crematoria	through smoking or exposure to secondhand smoke eating vegetables from polluted areas (cadmium accumulation in vegetables) inhalation of cadmium-laden dust	kidney function disruption increased risk of osteoporosis and bone fractures carcinogenic (mainly lung cancer)
Lead in blood; exposure measurement of previous 3–4 months	leaded paint leaded drinking water pipes ferrous and non-ferrous industry in the past: leaded petrol	inhalation of lead-contaminated dust in regions with historical lead pollution (e.g., near industry or busy roads) lead particles can settle on vegetables and can pollute drinking water	anemia negative influence on intelligence in children kidney function disruption fertility problems probably carcinogenic
Copper in blood; exposure measurement of previous days	copper mining landfill sites, waste incineration timber production fossil fuel combustion	inhalation of copper-contaminated dust in some regions, drinking water contains high concentrations of copper	low concentrations are essential for good health in case of prolonged, high exposure: headache, nausea, diarrhea, dizziness

**Table 3:** Demographic and socioeconomic characteristics of respondents.

Variable	Respondents Follow-Up Study		Participants HBM Study	
	*n*	%	*n*	%
Age				
≤25 Year	1	9.1	30	10.7
25–30 Year	4	36.4	111	39.5
30–35 Year	4	36.4	101	35.9
>35 Year	2	18.2	39	13.9
Educational attainment				
Low (ISCED 0–2)	0	0.0	26	9.3
Medium (ISCED 3–4)	3	27.3	88	31.3
High (ISCED 5–8)	8	72.7	166	59.1
Employment status				
Working	9	81.8	238	87.5
Not working	2	18.2	34	12.5
Equivalent income *				
<1.250 euro	2	18.2	59	24.5
1.250–2.000 euro	5	45.5	117	48.5
>2.000 euro	4	36.4	65	27.0
Home ownership				
No	2	18.8	78	27.9
Yes	9	81.8	202	72.1
Migrant background **				
No	7	63.6	220	78.3
Yes	4	36.4	57	20.3
Parity				
1	4	36.4	126	44.8
2	4	36.4	100	35.6
3 and above	3	27.3	55	19.6

Note * Equivalent income was calculated as the monthly household’s income divided by the number of household members. Note ** Migrant background was ascribed to respondents with at least one parent that was not a native-born Belgian.

**Table 4:** Common responses to the topic of study participation.

	Reasons for Participating in Biomonitoring Study
Common Responses	To make a contribution to scientific research Because environmental health is an important theme Curious about own results

**Table 5:** Common responses to the topic of receiving results and evaluation of report-back practice.

	Initial Feeling When Receiving Individual Results		
Common Responses	A sense of not understanding the results A feeling of concern because of an elevated value		
	**Evaluation of Report-Back Practice**		
	Personal Result Format	Background Information	Option to Consult with Study Physician
Common Responses and Questions	How to interpret results in safety terms? What about transfer of chemicals to baby? Need for a (visual) summary	Lack of practical advice Too much scientific terminology List of possible health effects caused unpleasant feeling	No recollection of this option Option was not inviting or accessible enough Feeling of not wanting to disturb

**Table 6:** Common responses to the topic of study impact.

	Responses to Receiving Individual Results
Common Responses	Searching additional information on the internet Taking personal action (e.g., drinking water analysis) No further steps taken

## Data Availability

The datasets generated and/or analyzed during the current study are not publicly available due to privacy restrictions but are available from the corresponding author upon reasonable request.

## Supplementary Materials

The following are available online at https://www.mdpi.com/article/10.3390/toxics9040069/s1: Table S1 Data format used for individual report-back of HBM-results (translated from Dutch) (2013–2015), Table S2: Background information for individual report-back of HBM results (translated from Dutch) (2013–2015); Supplemented materials: Info brochure for HBM recruitment (translated from Dutch), Introductory letter for personal results report (translated from Dutch), Follow-up interview guideline.

## References

[1] Needham L.L., Calafat A.M., Barr D.B. (2007). Uses and issues of biomonitoring. Int. J. Hyg. Environ. Health.

[2] WHO (2015). Human Biomonitoring: Facts and Figures.

[3] Choi J., Aarøe Mørck T., Polcher A., Knudsen L., Joas A. (2015). Review of the State of the Art of Human Biomonitoring for Chemical Substances and Its Application to Human Exposure Assessment for Food Safety. EFSA Support. Publ..

[4] Washburn R. (2013). The social significance of human biomonitoring. Sociol. Compass.

[5] Morello-Frosch R., Brody J.G., Brown P., Altman R.G., Rudel R.A., Pérez C. (2009). Toxic ignorance and right-to-know in biomonitoring results communication: A survey of scientists and study participants. Environ. Health.

[6] Ohayon J.L., Cousins E.M., Brown P., Morello-Frosch R., Brody J.G. (2017). Researcher and institutional review board perspectives on the benefits and challenges of reporting back biomonitoring and environmental exposure results. Environ. Res..

[7] Hintz E.A., Dean M. (2020). Best practices for returning research findings to participants: Methodological and ethical considerations for communication researchers. Commun. Methods Meas..

[8] Exley K., Cano N., Aerts D., Biot P., Casteleyn L., Kolossa-Gehring M., Schwedler G., Castano A., Angerer J., Koch H.M. (2015). Communication in a human biomonitoring study: Focus group work, public engagement and lessons learnt in 17 european countries. Environ. Res..

[9] Morello-Frosch R., Varshavsky J., Liboiron M., Brown P., Brody J.G. (2015). Communicating results in post-belmont era biomonitoring studies: Lessons from genetics and neuroimaging research. Environ. Res..

[10] Ramirez-Andreotta M.D., Brody J.G., Lothrop N., Loh M., Beamer P.I., Brown P. (2016). Improving environmental health literacy and justice through environmental exposure results communication. Int. J. Environ. Res. Public Health.

[11] Washburn R. (2013). Rethinking the disclosure debates: A situational analysis of the multiple meanings of human biomonitoring data. Crit. Public Health.

[12] Emmett E.A., Desai C. (2010). Community first communication: Reversing information disparities to achieve environmental justice. Environ. Justice.

[13] Louro H., Heinala M., Bessems J., Buekers J., Vermeire T., Woutersen M., van Engelen J., Borges T., Rousselle C., Ougier E. (2019). Human biomonitoring in health risk assessment in europe: Current practices and recommendations for the future. Int. J. Hyg. Environ. Health.

[14] Keune H., Morrens B., Loots I. (2008). Risk communication and human biomonitoring: Which practical lessons from the belgian experience are of use for the eu perspective?. Environ. Health.

[15] Middleton D.R., Watts M.J., Hamilton E.M., Fletcher T., Leonardi G.S., Close R.M., Exley K.S., Crabbe H., Polya D.A. (2016). Prolonged exposure to arsenic in uk private water supplies: Toenail, hair and drinking water concentrations. Env. Sci Process Impacts.

[16] Lind U., Mose T., Knudsen L.E. (2007). Participation in environmental health research by placenta donation-A perception study. Environ. Health.

[17] Purvis R.S., Abraham T.H., Long C.R., Stewart M.K., Warmack T.S., McElfish P.A. (2017). Qualitative study of participants’ perceptions and preferences regarding research dissemination. AJOB Empir Bioeth.

[18] Brody J.G., Morello-Frosch R., Brown P., Rudel R.A., Altman R.G., Frye M., Osimo C.A., Perez C., Seryak L.M. (2007). Improving disclosure and consent: “Is it safe?”: New ethics for reporting personal exposures to environmental chemicals. Am. J. Public Health.

[19] Altman R.G., Morello-Frosch R., Brody J.G., Rudel R., Brown P., Averick M. (2008). Pollution comes home and gets personal: Women’s experience of household chemical exposure. J. Health Soc. Behav..

[20] Adams C., Brown P., Morello-Frosch R., Brody J.G., Rudel R., Zota A., Dunagan S., Tovar J., Patton S. (2011). Disentangling the exposure experience: The roles of community context and report-back of environmental exposure data. J. Health Soc. Behav..

[21] Judge J.M., Brown P., Brody J.G., Ryan S. (2016). The exposure experience: Ohio river valley residents respond to local perfluorooctanoic acid (pfoa) contamination. J. Health Soc. Behav..

[22] Brody J.G., Dunagan S.C., Morello-Frosch R., Brown P., Patton S., Rudel R.A. (2014). Reporting individual results for biomonitoring and environmental exposures: Lessons learned from environmental communication case studies. Environ. Health.

[23] Dunagan S.C., Brody J.G., Morello-Frosch R., Brown P., Goho S., Tovar J., Patton S., Danford R. (2013). When Pollution is Personal: Handbook for Reporting Results to Participants in Biomonitoring and Personal Exposure Studies.

[24] Schoeters G., Govarts E., Bruckers L., Den Hond E., Nelen V., De Henauw S., Sioen I., Nawrot T.S., Plusquin M., Vriens A. (2017). Three cycles of human biomonitoring in flanders−time trends observed in the flemish environment and health study. Int. J. Hyg. Environ. Health.

[25] Colles A., Bruckers L., Den Hond E., Govarts E., Morrens B., Schettgen T., Buekers J., Coertjens D., Nawrot T., Loots I. (2019). Perfluorinated substances in the flemish population (Belgium): Levels and determinants of variability in exposure. Chemosphere.

[26] Reynders H., Colles A., Morrens B., Mampaey M., Coertjens D., Koppen G., Schoeters G., Loots I., Chovanova H., Winderickx W. (2017). The added value of a surveillance human biomonitoring program: The case of flehs in flanders (belgium). Int. J. Hyg. Environ. Health.

[27] Schoeters G., Den Hond E., Colles A., Loots I., Morrens B., Keune H., Bruckers L., Nawrot T., Sioen I., De Coster S. (2012). Concept of the flemish human biomonitoring programme. Int. J. Hyg. Environ. Health.

[28] Boronow K.E., Susmann H.P., Gajos K.Z., Rudel R.A., Arnold K.C., Brown P., Morello-Frosch R., Havas L., Brody J.G. (2017). Derbi: A digital method to help researchers offer “right-to-know” personal exposure results. Environ. Health Perspect..

[29] Rothstein M.A. (2006). Tiered disclosure options promote the autonomy and well-being of research subjects. Am. J. Bioeth..

[30] Buck A.J., Vena J.E., McGuinness B.M., Cooney M.A., Louis G.M. (2010). Communicating serum chemical concentrations to study participants: Follow up survey. Environ. Health.

[31] Halkoaho A., Pietilä A.-M., Dumez B., Van Damme K., Heinonen S., Vähäkangas K. (2010). Ethical aspects of human placental perfusion: Interview of the mothers donating placenta. Placenta.

[32] Perovich L.J., Ohayon J.L., Cousins E.M., Morello-Frosch R., Brown P., Adamkiewicz G., Brody J.G. (2018). Reporting to parents on children’s exposures to asthma triggers in low- income and public housing, an interview-based case study of ethics, environmental literacy, individual action, and public health benefits. Environ. Health.

[33] Claudio L., Gilmore J., Roy M., Brenner B. (2018). Communicating environmental exposure results and health information in a community-based participatory research study. BMC Public Health.

[34] Hernick A.D., Brown M.K., Pinney S.M., Biro F.M., Ball K.M., Bornschein R.L. (2011). Sharing unexpected biomarker results with study participants. Environ. Health Perspect..

