# Supplementary material for: Participant Experiences in a Human Biomonitoring Study: Follow-Up Interviews with Participants of the Flemish Environment and Health Study
Source: Toxics. 2021 Mar 28;9(4):69. doi: 10.3390/toxics9040069 (PMC8066005; doi:10.3390/toxics9040069)
Supplement: Supplementary file 1 [file toxics-09-00069-s001.zip › toxics-1139747 - supplem 3.pdf]

Subject: Personal results of the Flemish Human Biomonitoring Study on Environment and Health.

Dear Parents,

You have participated in a study that measures how many pollutants newborns have in their bodies. This study was carried out by the Steunpunt Milieu en Gezondheid (Environment and Health Support Centre), commissioned by the Flemish government. We hope that you and your child are doing well. Through this letter we would like to thank you again for your participation and inform you about the results.

What information do you find with this letter?

1. The result sheet with your personal values of pollutants and health measurements
2. A table with information about the measured pollutants
3. A summary of the group results of the study

What was the study about again?

During the period from November 2013 to November 2014, cord blood was collected from 281 newborns in 6 maternity hospitals in Flanders. A number of substances were measured in it. On the one hand, these are measurements that indicate the extent to which you have come into contact with environmental pollutants (e.g. lead, arsenic, PCBs). On the other hand, a number of measurements were carried out that provide information on the state of health (e.g. hormone levels).

The aim is to obtain more knowledge about the influence of the environment on health from the measurements of all newborns together. The main objective is to detect possible negative consequences of environmental pollution so that the government can adjust its policy on the basis of this knowledge.

Your personal results

On the results sheet we give the values of pollutants in your cord blood. You can compare your results with the group results of all participants together.

If one or more of your results require further follow-up, you have already been contacted by the study physician. If you have medical questions or would like a discussion of your results, you can contact Dr. Vera Nelen (03/259 12 90) on Thursday 17th December from 2 p.m. to 4 p.m. and Wednesday 23rd December from 2 p.m. to 4 p.m.

Attached to this letter you will find a table with more information about the substances that were measured. How are they created? How are we exposed and how can we reduce our own exposure? What are the health risks? You can also take a look at the website www.milieu-en-gezondheid.be.

Why is a result sometimes missing?

If there is no value filled in for a certain measurement, there is no measurement result for you. Occasionally a measurement is missing, e.g. because there was insufficient cord blood to perform the measurement in a quality manner.

What happens to the rest of the samples?

If there are leftovers from your samples, they are stored in the biobank. It is possible that they will be used to perform new measurements. For new measurements, permission is always sought from the ethics committee. All retained samples are coded anonymously. On the website www.milieu-en-gezondheid.be you can follow the results of new measurements through the research reports. The biobank is under supervision of the Flemish government after the conclusion of this biomonitoring campaign at the end of 2015.

Group results

You will also find a description of the main group results with this letter. To process the results at the group level (all participants), we used the answers from the questionnaires you completed. These answers were correlated with the measured values. You can request the full report with results via the form on the website www.milieu-en-gezondheid.be.

We are very grateful for your cooperation! By doing so, you help us to better understand the difficult relationship between environment and health.

Kind regards

Vera Nelen, medical doctor

Provincial Institute of Hygiene (PIH)

Kronenburgstraat 45

2000 Antwerp
