# Supplementary material for: Participant Experiences in a Human Biomonitoring Study: Follow-Up Interviews with Participants of the Flemish Environment and Health Study
Source: Toxics. 2021 Mar 28;9(4):69. doi: 10.3390/toxics9040069 (PMC8066005; doi:10.3390/toxics9040069)
Supplement: Supplementary file 1 [file toxics-09-00069-s001.zip › toxics-1139747 - supplem 4.pdf]

Steunpunt beleidsrelevant onderzoek 3de generatie

Milieu en Gezondheid

SCIENTIFIC RESEARCH ON ENVIRONMENT AND HEALTH

IN FLANDERS

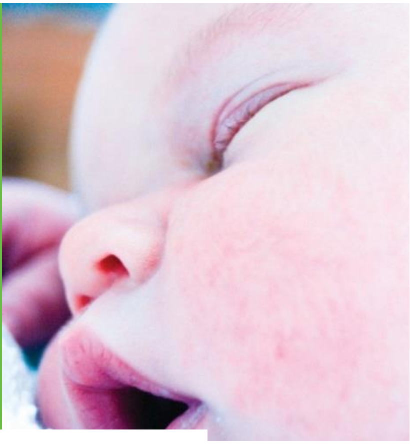

BIOMONITORING OF NEWBORNS

Want to know if your baby is growing up in a healthy environment?

Then participate in this measurement campaign, in which the Flemish government wants to find out whether environmental pollution in Flanders has an impact on health.

Researchers from various universities and research institutes want to find out whether traces of polluting substances from the environment can be found in the body. This type of research is called human biomonitoring.

Congratulations!

You have just brought a beautiful baby into the world.
You just gave birth and want to enjoy your baby most of all.
Right you are! But can we ask you a few questions?

Did you pay attention to a healthy lifestyle during pregnancy?

Do reports of harmful substances in toys or baby bottles ever worry you?
In short: Do you wonder if your child will grow up in a healthy environment?

Then please read on, because we would like to answer these questions together with you!

We would like to ask for your cooperation in a scientific study.

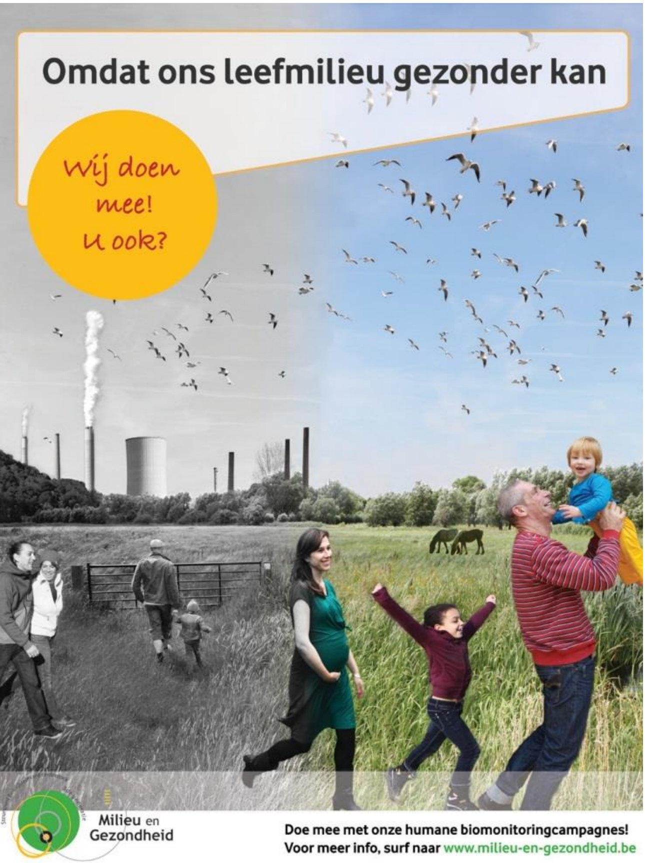

Who we are

Let's introduce:

'We' are scientific researchers from the five Flemish universities (Brussels, Ghent, Leuven, Antwerp and Hasselt) and 2 Flemish research institutes (VITO (Flemish Institution for Technological Research) and PIH (Provincial Institute for Hygiene)).

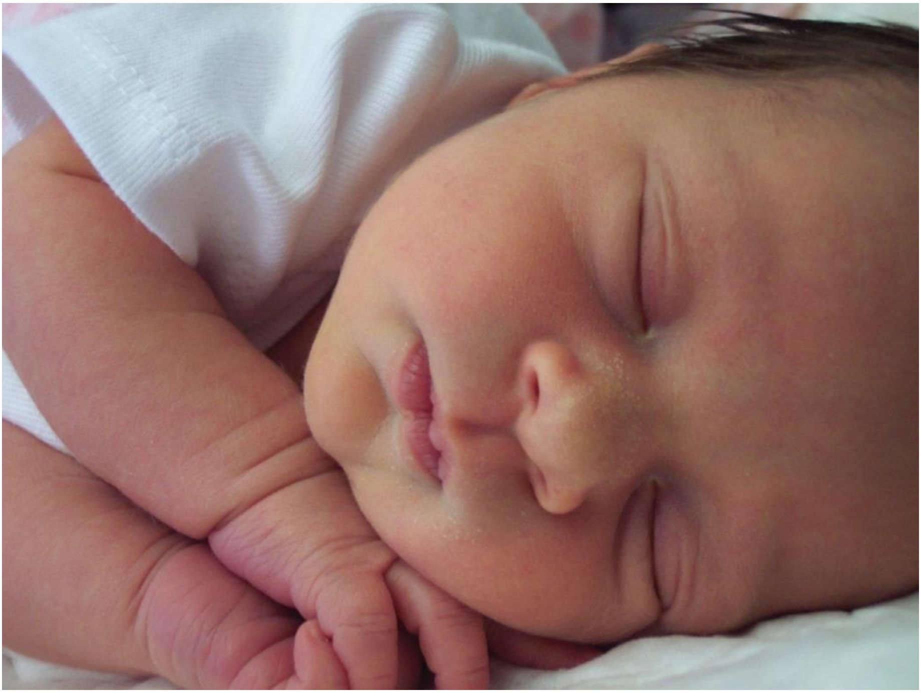

The study: what will we do?

Commissioned by the Flemish government, we are looking for people who want to help find out how the environment can affect health. In this study we investigate whether the substances that pollute our environment also end up in our bodies. This kind of research is called bio-monitoring (literally, measuring and monitoring in the body). We will visit 250 mothers in 5 maternities, who we will ask to participate in the study. In this way we want to get a picture of the pollution in Flanders.

What exactly do we want to measure?

1) Pollutants, with the scientific term: bio-markers of exposure. We can measure pollutants in hair and blood, among other things. The amount found reflects the degree to which the substance is present in your body. Technological progress has provided more comfort but unfortunately also new pollutants: e.g. plasticizers in plastic materials such as toys, substances in the non-stick coating of your tefal pan, preservatives in personal care products ....

2) But what we want to know above all is whether these pollutants in our bodies are disrupting our health. This is scientifically called 'bio-markers of effect'. Some examples: Does air pollution increase the risk of allergy? What do pesticides do to the hormone levels in your blood?

In short, we need your help to investigate the extent to which pollutants affect your health and that of your child.

What do we ask of you?

Information

In order to get a picture of your living environment, we will ask you to complete a questionnaire: e.g. about your living environment, eating habits, health, ... This will take about an hour. We also ask your permission to retrieve data about your baby from the medical records of the maternity clinic (birth weight, height, head circumference), from the center for metabolic diseases (heel prick) and from the Child and Family Karos database. It goes without saying that we treat the answers with complete discretion.

A sample

Umbilical cord blood: After delivery, the midwife clamps and cuts the umbilical cord. From the piece still attached to the placenta, she then collects a small amount (± 50 mL) of blood. This blood is normally lost. The examination does not affect your baby's health in any way.

Placenta: We ask the midwife to keep a piece of placenta.

A lock of hair: At her visit, our study nurse will ask for a small lock of hair (150 mg, this is about 1 teaspoon, to be cut off at the base of the neck). We will, of course, do this in a place that will have the least effect on your hairstyle.

Nails: Your nails reflect the environmental exposure you have come into contact with over the past few months. That's why we ask you to collect the nails you've cut for a month and send them to us.

If we see effects on health, the government will take measures. So you can help protect the health of all Flemish babies and their mothers!

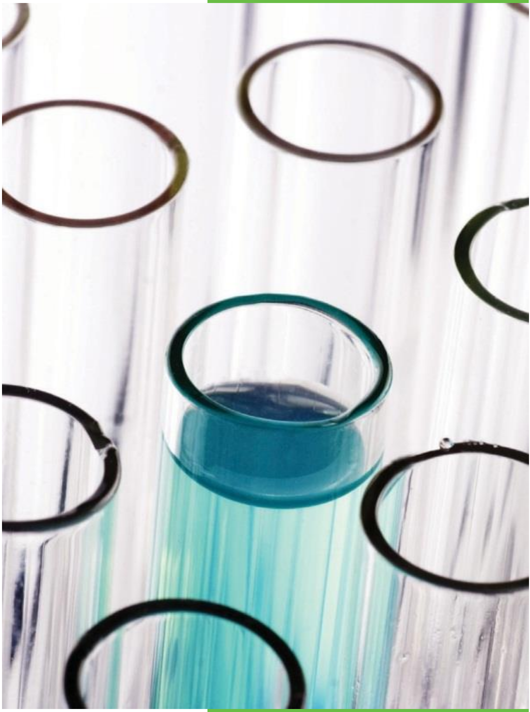

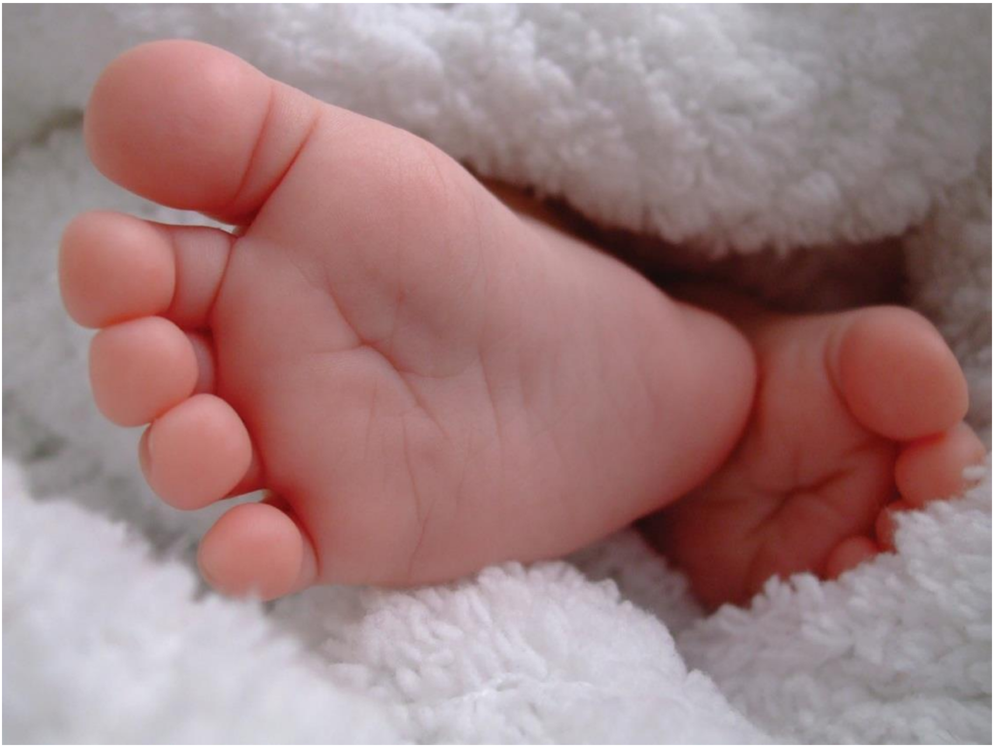

This research is done completely anonymous!

Your name or address will not be mentioned anywhere in the study. All samples and questionnaires are given a code.

What happens to the results of the study?

If you wish, you will receive the personal results of the analyses of your sample and your child's samples. The results are expected at the end of 2015.

You will also receive a report on the group results. Information about the status of the study can always be found on the website: www.milieu-en-gezondheid.be

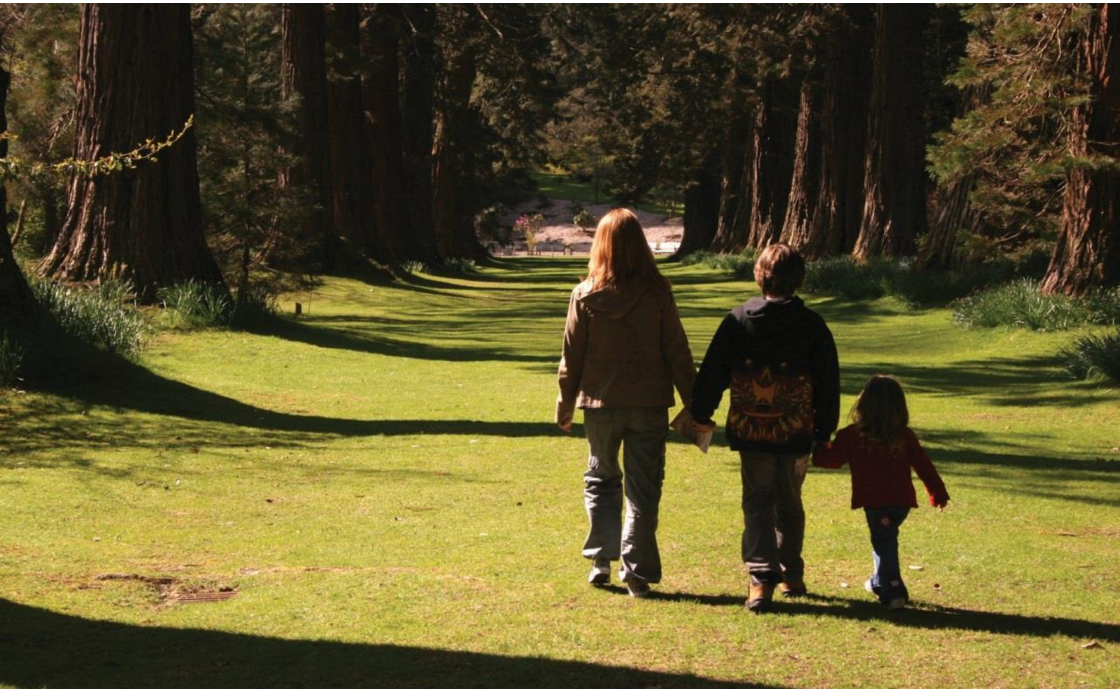

What happens to the remains of the samples?

Our knowledge about the environment is increasing every day and new measurement techniques are constantly being developed. Therefore, it is interesting to preserve the residual samples. In this way, we can measure new biomarkers in the future. Permission is always sought from the ethics committee for new analyses. All samples are coded and are only available to collaborators of the study.

At the end of the study (December 2015) you will be informed about the remaining samples: we will tell you what will happen to the samples and where you can go for information after 2015. This is under supervision of the Flemish government: Supervision Public Health (TOVO) and the Department of Environment, Nature and Energy (LNE).

How does it go from here?

If you would like to participate, we ask that you sign the attached consent form.

Questions?

Provincial Institute of Hygiene
Kronenburgstraat 45 – 2000 Antwerp

Physician: Vera Nelen
Phone: 03/259 12 61

Sociologist: Els Van de Mieroop
Phone: 03/259 12 61
e-mail: els.vandemieroop@pih.provant.be

Study commissioned by the Flemish Minister for the Environment, Joke Schauvliege, and the Flemish Minister for Health, Jo Vandeurzen.

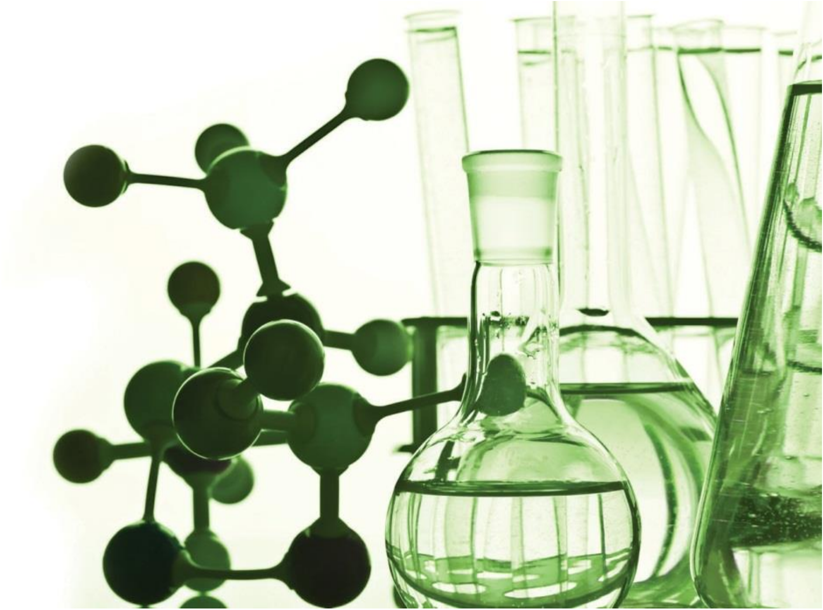

universiteit hasselt

KU LEUVEN

PROVINCIE ANTWERPEN

UNIVERSITEIT GENT

Universiteit Antwerpen

Vrije Universiteit Brussel

vito vision on technology
