# Supplementary material for: Participant Experiences in a Human Biomonitoring Study: Follow-Up Interviews with Participants of the Flemish Environment and Health Study
Source: Toxics. 2021 Mar 28;9(4):69. doi: 10.3390/toxics9040069 (PMC8066005; doi:10.3390/toxics9040069)
Supplement: Supplementary file 1 [file toxics-09-00069-s001.zip › toxics-1139747 - supplem 1.pdf]

toxics MDPI

Communication

Supplementary Material: Participant Experiences in a Human Biomonitoring Study: Follow-up Interviews with Participants of the Flemish Environment and Health Study

Bert Morrens, Hans Jonker, Elly Den Hond, Dries Coertjens, Ann Colles, Greet Schoeters, Nicolas Van Larebeke, Tim Nawrot, Adrian Covaci, Vera Nelen, Frédéric Vandermoere and Ilse Loots

Table S1. Data format used for individual report-back of HBM-results (translated from Dutch) (2013–2015).

Toxic Metals	Your Result	Results of All Participants	
		Median	P90
Cadmium (µg/L)	X	0.021	0.034
Lead (µg/L)	X	6.07	11.50
Copper (µg/L)	X	570.95	685.61
Manganese (µg/L)	X	30.15	46.25
Thallium (ng/L)	X	18.00	26.31
Arsenic (µg/L)	X	0.653	2.845
Methylmercury in hair (µg/L)	X	0.30	0.71
Chlorinated substances			
PCBs (ng/g lipid)	X	24.15	53.19
Metabolite of DDT (ng/g lipid)	X	55.39	143.89
Hexachlorobenzene (ng/g lipid)	X	14.82	27.16
Oxychlordane (ng/g lipid)	X	1.52	2.89
Trans-nonachlor (ng/g lipid)	X	ND	2.88
Lindane (ng/g lipid)	X	3.8	7.7
Perfluorinated compounds			
PFOS (µg/L)	X	1.11	2.56
PFOA (µg/L)	X	1.27	2.14
PFHxS (µg/L)	X	0.37	0.74
PFNA (µg/L)	X	0.21	0.44

Table 2. Background information for individual report-back of HBM results (translated from Dutch) (2013–2015).

Pollutants	What are the Main Sources in Our Environment?	How are Humans Exposed?	What are Possible Health Risks?
**Toxic Metals**			
Cadmium in blood; exposure measurement of previous 3–4 months	• cigarette smoke<br>• non-ferrous industry, scrap processing industry<br>• in the past: domestic waste incinerators (e.g., battery combustion) and crematoria	• through smoking or exposure to secondhand smoke<br>• eating vegetables from polluted areas (cadmium accumulation in vegetables)<br>• inhalation of cadmium-laden dust	• kidney function disruption<br>• increased risk of osteoporosis and bone fractures<br>• carcinogenic (mainly lung cancer)
Lead in blood; exposure measurement of previous 3–4 months	• leaded paint<br>• leaded drinking water pipes<br>• ferrous and non-ferrous industry<br>• in the past: leaded petrol	• inhalation of lead-contaminated dust<br>• in regions with historical lead pollution (e.g., near industry or busy roads) lead particles can settle on vegetables and can pollute drinking water	• anemia<br>• negative influence on intelligence in children<br>• kidney function disruption<br>• fertility problems<br>• probably carcinogenic

Copper in blood; exposure measurement of previous days	• copper mining<br>• landfill sites, waste incineration<br>• timber production<br>• fossil fuel combustion	• inhalation of copper-contaminated dust<br>• in some regions, drinking water contains high concentrations of copper	• low concentrations are essential for good health<br>• in case of prolonged, high exposure: headache, nausea, diarrhea, dizziness
Manganese in blood; exposure measurement of previous days	• Industry: metallurgy, chemical industry, glass production, leather and textile industry, fertilizers<br>• important component in welding	• inhalation of manganese-containing dust near industry<br>• when welding	• harmful to the nervous system
Thallium in blood; exposure period not known	• cigarette smoke<br>• electronics industry, mineral melting furnaces, coal-fired power plants, cement and brick industries	• through smoking or exposure to secondhand smoke<br>• fruits and vegetables grown near industries that process thallium	• prolonged exposure is harmful to the nervous system, heart, lungs, liver and kidneys
Arsenic in blood; measure of recent exposure (1–2 days)	• wood treatment<br>• batteries, conductors, diodes<br>• in the past: used as a pesticide, this is currently prohibited	• the amount of total arsenic reflects the toxic and non-toxic arsenic and is strongly influenced by the non-toxic form<br>• fish is an important source of arsenic, but this is the non-toxic form of arsenic<br>• exposure to toxic arsenic occurs through water, food and through the skin	• carcinogenic<br>• skin diseases and skin irritation
Methylmercury (the toxic form of mercury) in hair	• thermometers, mercury vapor lamps, batteries, electric switches<br>• amalgam tooth fillings<br>• control of fungi	• fish is a major source of mercury<br>• amalgam dental fillings release mercury in saliva<br>• low exposure through air, water and food	• harmful to the nervous system<br>• disruption of kidney function
Chlorinated substances			
PCBs (138, 153, 180) in blood; exposure measurement of last 10–20 years	• currently prohibited<br>• in the past massively used in transformers, capacitors, paints, inks, insulating materials	• due to accidents and mistakes, PCBs have contaminated the food chain; since they are fat-soluble, they are mainly found in fatty foods (fatty fish, meat, dairy products)<br>• PCBs may occur in the air near incinerators, crematoria	• endocrine disruptor; adverse effect on fertility<br>• probably carcinogenic<br>• harmful to the nervous system<br>• disruption of the immune system
Metabolite of DDT in blood; exposure measurement of recent years	• insecticide<br>• banned in Belgium since the 1970s but still allowed in countries with malaria	• DDT is fat-soluble and has accumulated in the food chain (especially high-fat foods like fatty fish, meat, dairy)<br>• Via water, soil, dust, humans come into contact with DDT residues from historical pollution	• endocrine disruptor; adverse effect on fertility<br>• possibly carcinogenic<br>• harmful to the nervous system<br>• disruption of the immune system
HCBB	• currently prohibited	• HCB is fat-soluble and has accumulated in the food chain	• endocrine disruptor; adverse effect on fertility

in blood; exposure measurement of recent years	• fungicide for plants, seeds, cereals<br>• in the past: used in manufacture of fireworks, ammunition and rubber	(especially high-fat foods such as fatty fish, meat, dairy).<br>• through water, soil, dust, humans come into contact with residues of HCB from historical pollution	• possibly carcinogenic<br>• harmful to the nervous system<br>• disturbance of the immune system
Chlordanes (oxychlordane, trans-nonachlor) in blood; exposure measurement of recent years	• insecticides on cereals, citrus fruits and in private lawns and gardens<br>• Prohibited in Belgium for agricultural applications since 1981, for all other applications since 1998	• through water, soil, dust, humans come in contact with chlordane from historical pollution<br>• chlordane accumulates in the food chain (fish, birds and mammals)	• harmful to the nervous system<br>• endocrine disruptor<br>• possibly carcinogenic
Hydrophobic perfluorinated compounds			
PFOS<br>PFOA<br>PFHxS<br>PFNA<br>in blood; exposure measurement of recent years	• used to make products water-, and grease-repellent, including tefal pans, cardboard drinking cups, furniture upholstery, carpets,...<br>• used in fire extinguishers, cleaning products, cosmetics, film materials, ...	• perfluorinated food gets into the food through the use of (damaged) tefal pans, drinking cups, etc.<br>• perfluorinated material can bind to dust particles (e.g., from carpets, furniture,...) and can be inhaled by humans.<br>• perfluorinated products accumulate in the food chain (especially fish)	• endocrine disruptor; negative impact on fertility<br>• harmful to the nervous system<br>• harmful to the heart and blood vessels
