# Supplementary material for: Participant Experiences in a Human Biomonitoring Study: Follow-Up Interviews with Participants of the Flemish Environment and Health Study
Source: Toxics. 2021 Mar 28;9(4):69. doi: 10.3390/toxics9040069 (PMC8066005; doi:10.3390/toxics9040069)
Supplement: Supplementary file 1 [file toxics-09-00069-s001.zip › toxics-1139747 - supplem 2.pdf]

Supplemented material: Interview guideline

1. **Questions about study participation**
- How did you get in touch with the study? (where and when)
- What was your first reaction when you heard about the investigation?
- When did you decide to participate? Immediately or did you need time to think about it?
- Why did you participate? What kind of motivation was there?
- Have you talked to others about study participation? If so, what was their reaction?
- Have you participated in other research in the past? If so, what kind of study was that?
- How did you experience the study? What did you like and dislike?
- What were your expectations at the start of the study and were they met at the end of the study?
- Would you participate in the study again? Are there parts of the study that you would have preferred to do differently?
- What benefits did you experience for yourself by participating in this study?
- What disadvantages did you experience for yourself by participating in this study?
- In your opinion, how could we increase the degree of participation in our study?

2. **Questions about study results**
- Personal results
    - Do you remember how the results made you feel? (were you worried or not?)
    - What do you remember about your results? (did you keep the results letter?)
    - How did you interpret your results? What reference did you use to compare your own values? How did you interpret the reference values (does the mean value feel like a safe limit to you?).
    - Would a graphical visualization the results be useful? (traffic light representation)
    - Did you find the letter comprehensible? Did you have any comments on the wording or on the way the letter was drafted?
    - Was there sufficient background information? would you rather have more information (and if so, what type of information) or less? Should information reach you in a more phased way?
    - Would a personal conversation with a study doctor be useful?
    - Have you searched for extra information yourself, e.g. via the internet?
    - Have you discussed the results with someone?
    - How important is the privacy of your personal results to you? (e.g.: presentation on card defensible? Passing on data to third parties? Showing participant status?)
- Group results (the letter with summary of the study results)
    - Have you read the letter with the group results of all participants together? What did you remember about that?
    - Imagine that for a certain substance your personal value is low, but that that substance is still a problem for the Flemish population in general. Would this keep you awake at night?

3. Questions about study impact

- Have you taken certain actions or made changes as a result of the results? E.g. in purchasing behavior, in nutrition, in lifestyle? Or follow-up through doctors?
- Do you feel you can now make more informed choices about how to avoid or reduce exposure?
- Did the results make you look differently at your body and your health?
- Do you experience (news) reports about environmental pollution differently than before the study? Do you now look at the problem in a different way?
- In general, are you more or less worried about the impact of environmental pollution than before the study? (Are you happy to know your personal values?)
- Where do you stand on environmental health research?
- Who do you think should tackle the risks associated with environmental pollution in particular?
- Based on this study, are there any messages you would like to give to your children?
- Are there messages that you think everyone should know about?

Are there any issues related to the study that you would like to mention? Or suggestions you would like to make to the research team?
